# Radiotherapy improves survival in HER2-positive breast cancer with lung metastases: a retrospective study with artificial intelligence-based prognostic modeling

**DOI:** 10.3389/fonc.2025.1633448

**Published:** 2025-07-16

**Authors:** Tengfei Ren, Xiangkun Wang, Shengyuan Luo, Shanbao Ke

**Affiliations:** ^1^ Department of Hemangioma, Henan Provincial People’s Hospital, Zhengzhou University People’s Hospital, Henan University People’s Hospital, Zhengzhou, China; ^2^ Department of Breast and Thyroid Surgery, Juye County People’s Hospital, Heze, Shandong, China; ^3^ Department of The Second Clinical Medical College, Changzhi Medical College, Changzhi, China; ^4^ Department of Breast Center, West China Hospital, Sichuan University, Chengdu, China; ^5^ Department of Oncology, Henan Provincial People’s Hospital, Zhengzhou University People’s Hospital, Henan University People’s Hospital, Zhengzhou, China

**Keywords:** radiotherapy, survival, breast cancer, lung metastases, artificial intelligence

## Abstract

**Background:**

Human epidermal growth factor receptor 2 (HER2)-positive breast cancer is an aggressive subtype with a high risk of distant metastasis, particularly to the lungs. While systemic therapies have improved outcomes, the role of radiotherapy (RT) in the management of lung metastases remains uncertain.

**Methods:**

This retrospective study analyzed 248 HER2-positive breast cancer patients with lung metastases treated at two institutions between 2006 and 2021. Propensity score matching (PSM) was used to balance baseline characteristics between the RT and non-RT groups. Overall survival (OS) was assessed using Kaplan–Meier curves and Cox regression. A least absolute shrinkage and selection operator (LASSO)-Cox model was developed to identify prognostic factors, and its performance was evaluated using risk score visualization, receiver operating characteristic (ROC) analysis, and decision curve analysis (DCA).

**Results:**

RT significantly improved median OS both before (50.4 vs. 34.0 months, p < 0.001) and after PSM (51.5 vs. 32.3 months, p < 0.001). LASSO-Cox analysis confirmed RT as an independent prognostic factor. The predictive model demonstrated good discrimination (1- and 3-year AUCs of 0.716 and 0.722, respectively) and clinical utility by DCA.

**Conclusion:**

RT offers a significant survival benefit in HER2-positive breast cancer patients with lung metastases. AI-based modeling enhances prognostic accuracy and supports personalized treatment decisions.

## 1 Introduction

Human epidermal growth factor receptor 2 (HER2)-positive breast cancer is a biologically aggressive subtype accounting for approximately 15–20% of all breast cancer cases (1). It is characterized by high rates of proliferation and a strong tendency for distant metastasis, particularly to the lung (2). Systemic therapies including trastuzumab, pertuzumab, T-DM1, and trastuzumab deruxtecan have significantly extended survival in HER2-positive breast cancer (3, 4). However, controlling pulmonary metastases continues to pose a clinical challenge, particularly in the context of symptomatic or oligometastatic disease.

Radiotherapy (RT) has become an important adjunctive treatment for such patients, offering local control and symptom relief (5). However, the role of RT in HER2-positive breast cancer with lung metastasis is still not clearly defined due to heterogeneity in tumor burden, treatment timing, and patient response (6). In recent years, artificial intelligence (AI) has emerged as a powerful tool in oncologic decision-making, with applications ranging from radiomics-based lesion characterization to individualized treatment prediction (7–9).

Radiomics and machine learning models can extract high-dimensional features from CT or PET/CT images, enabling the non-invasive prediction of tumor biology, radiosensitivity, and treatment outcomes (10). These AI-driven methods offer the potential to identify patients who are most likely to benefit from RT, optimize radiation planning, and monitor therapeutic response in real time (11). In HER2-positive metastatic breast cancer, where treatment strategies must balance systemic control with local interventions, AI can support a more personalized, data-driven approach (12).

Despite these advances, few studies have specifically explored the integration of AI tools in guiding RT for lung metastases in HER2-positive breast cancer. Further research is warranted to validate predictive models and establish their clinical utility. A multidisciplinary strategy combining targeted systemic therapy, precise radiotherapy, and AI-based decision support may represent a promising direction to improve patient outcomes.

## 2 Methods

### 2.1 Characteristics of the cohort

This retrospective study enrolled a total of 248 patients with HER2-positive breast cancer and lung metastases who received treatment at two institutions between January 2006 and December 2021. HER2 positivity was defined according to ASCO/CAP guidelines as either immunohistochemistry (IHC) 3+, or IHC 2+ with confirmatory HER2 gene amplification by fluorescence *in situ* hybridization (FISH). HER2 testing protocols were standardized across both institutions. In cases with IHC 2+, reflex FISH testing was routinely performed to confirm HER2 status. Only patients with confirmed HER2 positivity were included in the study. Lung metastases were primarily diagnosed based on imaging modalities, including chest CT and PET/CT, interpreted by experienced radiologists. Histopathologic confirmation via biopsy was performed in selected cases when imaging results were inconclusive or clinically ambiguous. This study was approved by the Ethics Committee of West China Hospital, Sichuan University. At Henan Provincial People’s Hospital, ethical approval was waived due to the retrospective design and the use of anonymized patient data.

Inclusion criteria:

(1) histologically confirmed HER2-positive breast cancer; (2) presence of lung metastases confirmed by imaging or pathology; (3) complete and available clinical data.

Exclusion criteria:

(1) age under 18 years; (2) male sex; (3) overall survival less than 3 months; and (4) severe comorbidities making the patient unsuitable for radiotherapy.

### 2.2 Treatment method

All patients underwent first-line treatment with a combination of targeted therapy and chemotherapy following established protocols outlined in authoritative guidelines such as those issued by the NCCN and CSCO. Patients received intensity-modulated radiotherapy (IMRT), enabling conformal dose distribution and reduced toxicity. The median radiation dose delivered was 42 Gy. Treatment decisions were influenced by factors such as tumor burden, symptom control, the patient’s overall clinical status, and treatment costs.

### 2.3 Follow-up

The primary endpoint of this study were overall survival (OS).

OS was defined as the time from treatment initiation to death from any cause or last follow-up.

### 2.4 Statistical method

All statistical analyses were conducted using R software. Categorical variables were compared using the Chi-square test. For continuous variables such as age, normality was assessed using the Shapiro–Wilk test. Variables with a normal distribution were compared using the independent samples t-test, while non-normally distributed variables were analyzed using the Mann–Whitney U test. Propensity score matching (PSM) was applied to minimize baseline differences between the radiation and non-radiation groups. The PSM model included the following baseline covariates: age, hormone receptor status, Ki-67 index, T stage, N stage, targeted therapy, hormone therapy, and chemotherapy. Kaplan-Meier survival curves were generated to evaluate OS, and differences between groups were assessed using the log-rank test.

To identify potential prognostic factors associated with OS, we applied least absolute shrinkage and selection operator (LASSO) regression using 10-fold cross-validation. The optimal regularization parameter (λ) was chosen based on the minimum mean cross-validated partial likelihood deviance (λ.min), favoring a model with optimal predictive accuracy. Variables with non-zero coefficients at λ.min were retained and subsequently included in a multivariable Cox proportional hazards model to determine independent prognostic indicators. To stratify patients into high- and low-risk groups based on model-derived risk scores, we used the optimal cutoff point determined by maximally selected rank statistics. The risk score visualization plot (ggrisk plot), receiver operating characteristic (ROC) curves, and decision curve analysis (DCA) were used to evaluate the prognostic performance of the model.

## 3 Results

### 3.1 Study selection and characteristics

Baseline characteristics of the 248 HER2-positive breast cancer patients before PSM are summarized in **Table 1**. There were no statistically significant differences in age, hormone receptor status, Ki-67 index, T stage, N stage, or chemotherapy regimens between the radiotherapy and non-radiotherapy groups (all p > 0.05). The proportion of patients receiving trastuzumab combined with pertuzumab tended to be higher in the radiotherapy group (19.4% vs. 11.3%), although the difference did not reach statistical significance (p = 0.083). Hormone therapy showed a borderline difference between groups (p = 0.054), with the radiotherapy group having a higher proportion of hormone receptor-positive patients (48.4% vs. 35.5%).

**Table 1:** Baseline characteristics of HER2-positive breast cancer patients with or without radiotherapy before propensity score matching.

Variable	All patients (N=248)	No radiation (N=124)	Radiation (N=124)	p-value
Age, years (mean ± SD)	48.0 ± 11.1	48.2 ± 11.4	47.8 ± 10.8	0.798
Age group, n (%)				1
≤ 50	129 (52.0%)	64 (51.6%)	65 (52.4%)	
> 50	119 (48.0%)	60 (48.4%)	59 (47.6%)	
Hormone receptor status, n (%)				0.785
Negative	79 (31.9%)	41 (33.1%)	38 (30.6%)	
Positive	169 (68.1%)	83 (66.9%)	86 (69.4%)	
Ki-67 index, n (%)				1
< 14%	44 (17.7%)	22 (17.7%)	22 (17.7%)	
≥ 14%	204 (82.3%)	102 (82.3%)	102 (82.3%)	
T stage, n (%)				0.462
T1	24 (9.7%)	15 (12.1%)	9 (7.3%)	
T2	113 (45.6%)	58 (46.8%)	55 (44.4%)	
T3	58 (23.4%)	28 (22.6%)	30 (24.2%)	
T4	53 (21.4%)	23 (18.5%)	30 (24.2%)	
N stage, n (%)				0.215
N0	31 (12.5%)	19 (15.3%)	12 (9.7%)	
N1	70 (28.2%)	39 (31.5%)	31 (25.0%)	
N2	69 (27.8%)	33 (26.6%)	36 (29.0%)	
N3	78 (31.5%)	33 (26.6%)	45 (36.3%)	
Targeted therapy, n (%)				0.083
Trastuzumab	164 (66.1%)	90 (72.6%)	74 (59.7%)	
Trastuzumab + pertuzumab	38 (15.3%)	14 (11.3%)	24 (19.4%)	
Other	46 (18.5%)	20 (16.1%)	26 (21.0%)	
Chemotherapy, n (%)				0.916
Taxane	173 (69.8%)	89 (71.8%)	84 (67.7%)	
Anthracycline	4 (1.6%)	2 (1.6%)	2 (1.6%)	
Pyrimidine analog	62 (25.0%)	29 (23.4%)	33 (26.6%)	
Other	9 (3.6%)	4 (3.2%)	5 (4.0%)	
Hormone therapy, n (%)				0.054
Negative	144 (58.1%)	80 (64.5%)	64 (51.6%)	
Positive	104 (41.9%)	44 (35.5%)	60 (48.4%)	

After PSM, there was no significant difference in baseline characteristics between the two groups (**Table 2**).

**Table 2:** Baseline characteristics of HER2-positive breast cancer patients with or without radiotherapy after propensity score matching.

Variable	No radiotherapy (N=97)	Radiotherapy (N=97)	p-value
Age, years (mean ± SD)	48.85 ± 11.07	48.38 ± 10.24	0.762
Age group, n (%)			1
≤ 50	47 (48.5%)	47 (48.5%)	
> 50	50 (51.5%)	50 (51.5%)	
Hormone receptor status, n (%)			0.281
Negative	35 (36.1%)	27 (27.8%)	
Positive	62 (63.9%)	70 (72.2%)	
Ki-67 index, n (%)			0.715
< 14%	20 (20.6%)	17 (17.5%)	
≥ 14%	77 (79.4%)	80 (82.5%)	
T stage, n (%)			0.902
T1	8 (8.2%)	9 (9.3%)	
T2	46 (47.4%)	41 (42.3%)	
T3	23 (23.7%)	24 (24.7%)	
T4	20 (20.6%)	23 (23.7%)	
N stage, n (%)			0.988
N0	10 (10.3%)	10 (10.3%)	
N1	24 (24.7%)	24 (24.7%)	
N2	31 (32.0%)	29 (29.9%)	
N3	32 (33.0%)	34 (35.1%)	
Targeted therapy, n (%)			0.667
Trastuzumab	65 (67.0%)	60 (61.9%)	
Trastuzumab + pertuzumab	14 (14.4%)	14 (14.4%)	
Other	18 (18.6%)	23 (23.7%)	
Chemotherapy, n (%)			0.827
Taxane	69 (71.1%)	63 (64.9%)	
Anthracycline	2 (2.1%)	2 (2.1%)	
Pyrimidine analog	22 (22.7%)	27 (27.8%)	
Other	4 (4.1%)	5 (5.2%)	
Hormone therapy, n (%)			1
Negative	55 (56.7%)	55 (56.7%)	
Positive	42 (43.3%)	42 (43.3%)	

### 3.2 Overall survival before and after propensity score matching

**Figure 1:**
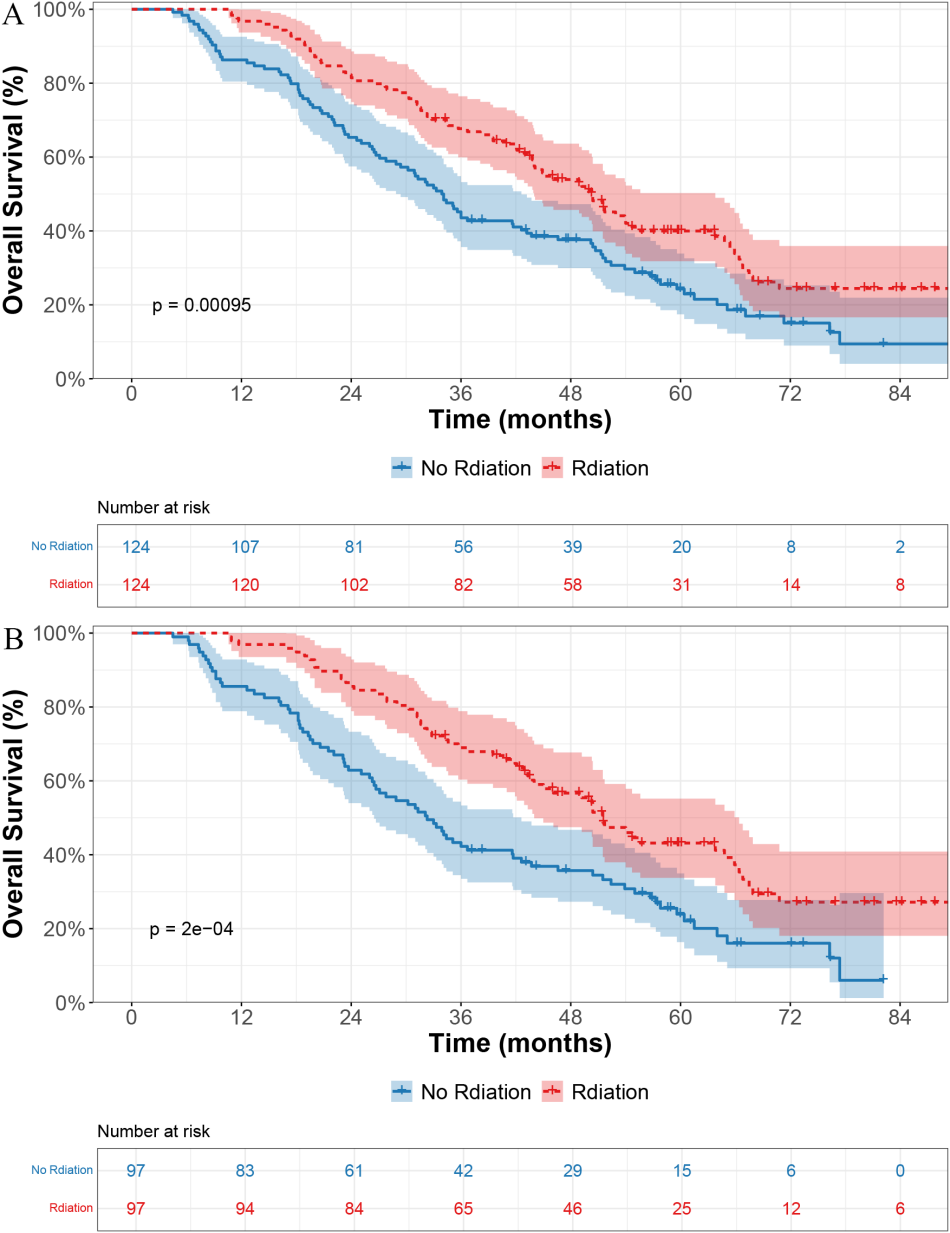
Kaplan–Meier curves for overall survival in patients receiving radiotherapy versus no radiotherapy. **(A)** Before propensity score matching (PSM). **(B)** After PSM.

As of the last follow-up, 80 of the 124 patients (64.5%) in the radiotherapy group and 98 of the 124 patients (79.0%) in the non-radiotherapy group had died. Before PSM, the median OS (mOS) was 50.4 months in the radiotherapy group and 34.0 months in the non-radiotherapy group, with a statistically significant difference (p < 0.001; **Figure 1A**).

After PSM, the survival benefit of radiotherapy remained evident, with a mOS of 51.5 months versus 32.3 months in the non-radiotherapy group (p < 0.001; **Figure 1B**).

### 3.3 Development and validation of AI models

**Figure 2:**
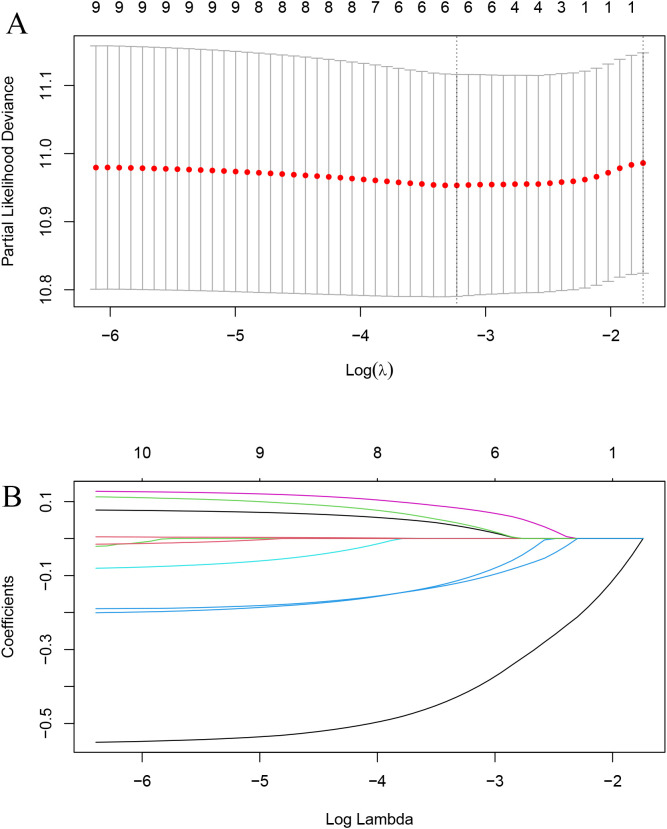
LASSO regression for variable selection. **(A)** Selection of optimal λ (lambda) using 10-fold cross-validation. **(B)** LASSO coefficient profiles of the variables.

**Figure 3:**
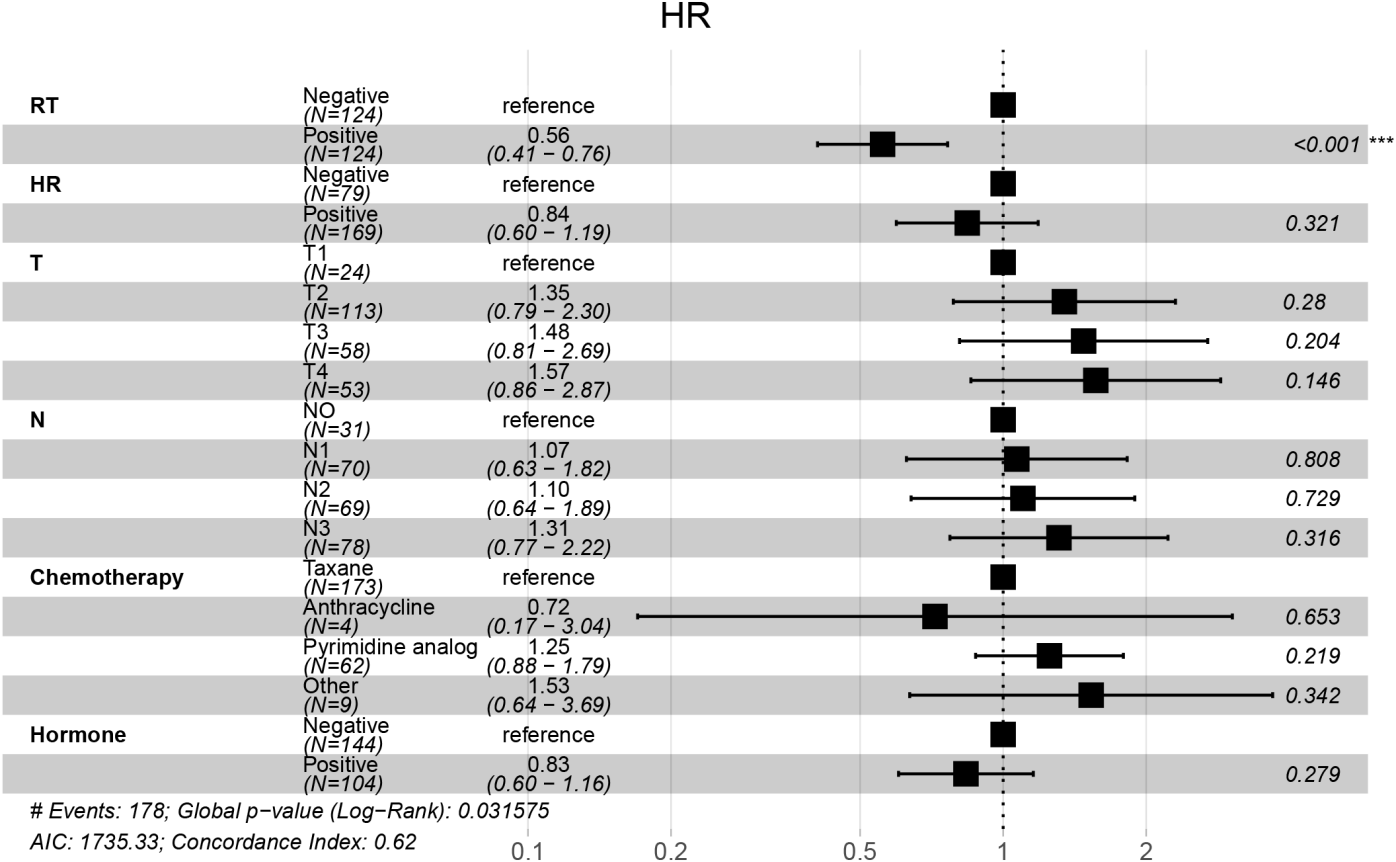
Forest plot of multivariable Cox regression for overall survival. Multivariable Cox analysis showing hazard ratios (HRs), 95% confidence intervals (CIs), and p-values for each clinical factor. Radiotherapy (RT) was significantly associated with improved overall survival.

LASSO regression identified RT, hormone receptor status, T stage, N stage, chemotherapy, and hormone therapy as prognostic factors for OS (**Figures 2A, B**). Subsequent multivariate Cox regression analysis confirmed that RT was an independent prognostic factor for OS (**Figure 3**).

**Figure 4:**
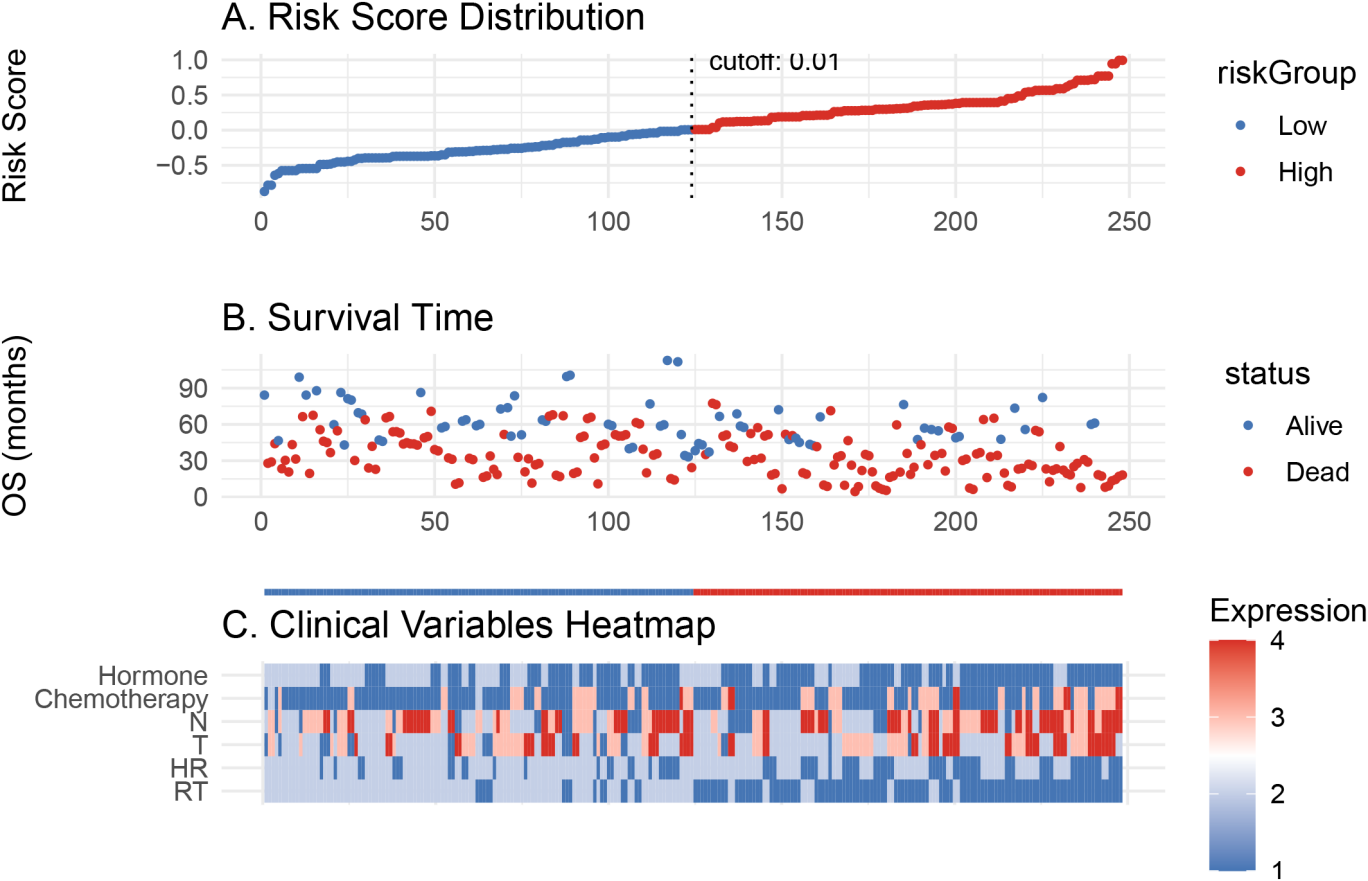
Risk score model evaluation and clinical correlation. **(A)** Risk score distribution of patients. **(B)** Overall survival time distribution for low- and high-risk groups. **(C)** Heatmap of clinical variable expression between the two risk groups.

Based on the Cox model, a risk score was calculated for each patient. Patients were divided into high- and low-risk groups using an optimal cutoff (cutoff = 0.01, **Figure 4A**). As shown in **Figure 4B**, patients in the high-risk group had more deaths and shorter OS. The heatmap in **Figure 4C** highlights that the distribution of RT differed between the two groups.

**Figure 5:**
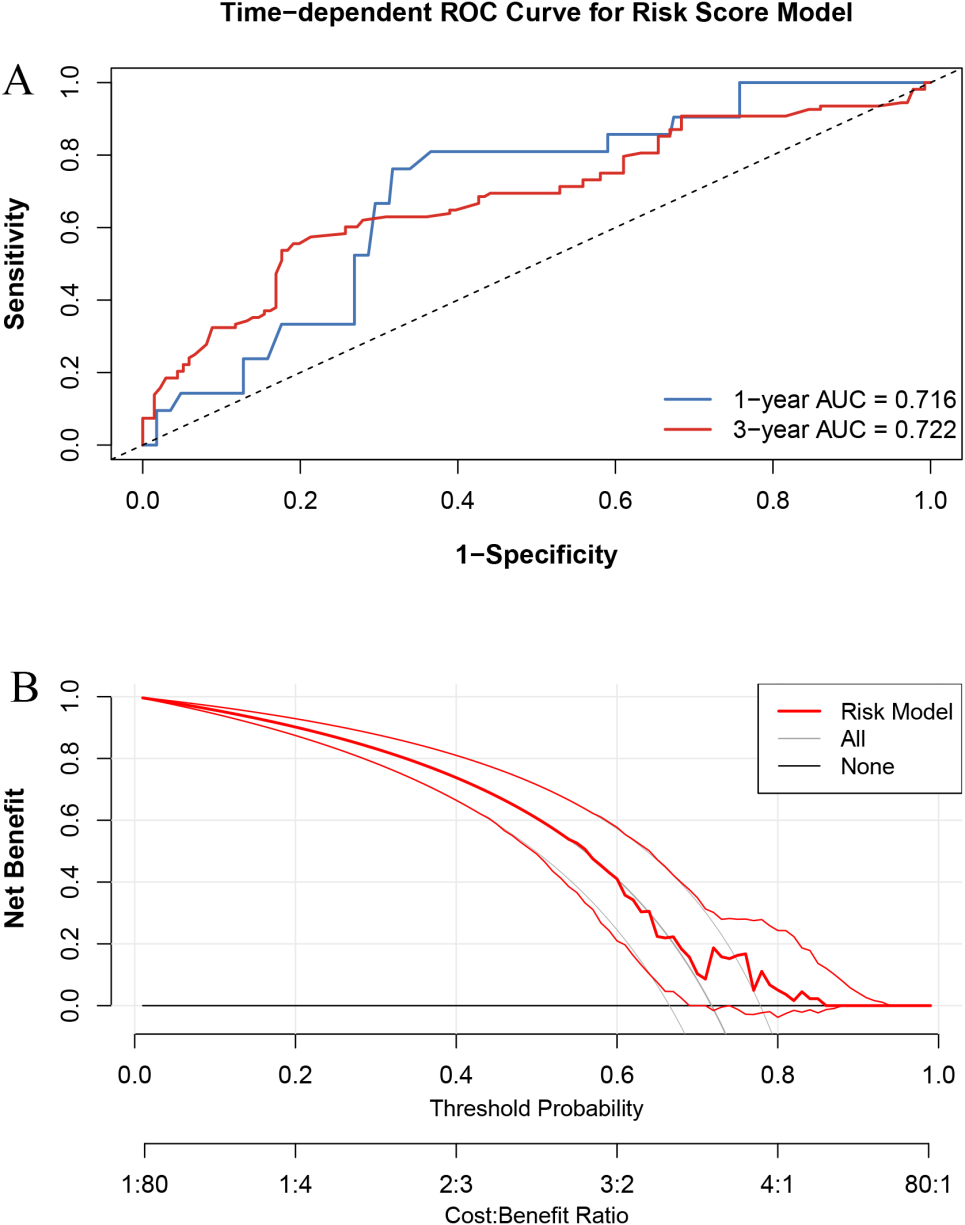
Performance evaluation of the risk score model. **(A)** Time-dependent ROC curves at 1-year and 3-year overall survival. **(B)** Decision curve analysis (DCA) comparing net benefit across threshold probabilities.

The ROC curves (**Figure 5A**) confirmed that the Cox model had good discriminatory ability, with AUCs of 0.716 and 0.722 at 1 and 3 years, respectively. The DCA (**Figure 5B**) further demonstrated the clinical usefulness and stability of the model.

## 4 Discussion

Patients with HER2-positive breast cancer represent a unique subgroup characterized by high aggressiveness and an increased propensity for visceral metastases, particularly to the lungs (13). Although the widespread application of HER2-targeted therapies such as trastuzumab and pertuzumab has significantly prolonged survival, the prognosis for patients with pulmonary metastatic disease remains unsatisfactory (14). This is particularly true when systemic control alone proves insufficient due to tumor heterogeneity or therapeutic resistance (15). Our findings provide compelling evidence supporting the integration of RT into the treatment paradigm for HER2-positive breast cancer patients with lung metastases.

This study revealed that patients who received RT experienced significantly prolonged OS compared to those who did not, both before and after PSM. The median OS benefit of over 16 months in the RT group suggests that local control through RT can offer meaningful clinical advantages, even in a metastatic context. Given the heterogeneity in metastatic burden and the existence of oligometastatic states where local interventions may result in long-term disease control, the inclusion of RT as part of multimodal therapy becomes particularly relevant. This survival advantage supports the growing body of evidence that aggressive local therapy may translate into improved systemic outcomes, particularly when combined with ongoing HER2-targeted treatments (14, 16).

Furthermore, this study incorporated AI-based modeling strategies to identify and validate key prognostic factors affecting OS. Using a LASSO regression followed by multivariate Cox analysis, we constructed a robust predictive model that included RT, hormone receptor status, T stage, N stage, chemotherapy, and hormone therapy. The predictive capacity of this model was confirmed by ROC analysis and DCA, demonstrating both strong discriminative ability and clinical applicability.

The application of AI in oncology, particularly in advanced disease settings, enables the processing of high-dimensional clinical and imaging data that may not be easily interpretable by traditional methods (17, 18). In our study, the integration of machine learning facilitated risk stratification and enabled visualization of individual patient profiles using tools such as the ggrisk plot and heatmaps. The ggrisk plot enables clinicians to visually assess each patient’s individual risk score as derived from the multivariable Cox model. Patients are stratified into high- and low-risk groups, allowing clinicians to quickly identify individuals with poorer predicted survival and potentially consider more aggressive or individualized interventions, such as consolidative radiotherapy or closer follow-up. This approach revealed a clear trend in which patients with favorable prognostic features, such as lower T and N stages and access to RT, were more likely to fall within the low-risk group (19). Such insights are invaluable for personalized treatment planning, especially in cases where the balance between systemic and local treatment needs to be carefully tailored (20).

Moreover, the AI-based risk model developed in this study holds practical potential for real-world clinical application. It can assist oncologists in stratifying patients with lung metastatic HER2-positive breast cancer into distinct prognostic groups, thereby informing treatment decisions (21). For instance, high-risk patients identified by the model may be considered for more aggressive strategies, including intensified radiotherapy, closer surveillance, or combination regimens. Conversely, low-risk patients may benefit from de-escalated treatment, minimizing unnecessary toxicity. Integrating this model into multidisciplinary workflows could help personalize care, optimize resource allocation, and enhance overall treatment efficacy. Such application is particularly valuable in complex metastatic cases where individualized decision-making is critical (22–25).

Despite the encouraging findings, several limitations must be acknowledged. First, the retrospective nature of the study may introduce selection bias and restrict control over potential confounding variables. Second, although our AI-based prognostic model demonstrated good performance in internal validation, it was not externally validated using an independent cohort, thereby limiting its generalizability. Future research should aim to incorporate external validation cohorts to confirm the model’s robustness. Additionally, the integration of radiomics, genomics, and immune profiling could further enhance the predictive power of the model and better reflect the biological complexity of metastatic HER2-positive breast cancer. Real-world prospective data will also be essential to validate and refine AI-assisted decision-making frameworks in radiotherapy.

## 5 Conclusions

In summary, our study demonstrates that RT is associated with a significant survival benefit in HER2-positive breast cancer patients with pulmonary metastases and underscores the potential of AI-based prognostic models to support more individualized and effective treatment strategies. The combination of traditional clinical parameters with advanced analytical tools represents a promising direction for optimizing care in this challenging population.

## Data Availability

The raw data supporting the conclusions of this article will be made available by the authors, without undue reservation.
